# Temporal dynamics and forecasting of respiratory viral infections during and after the SARS-CoV-2 pandemic (2020–2027): a multiplex PCR and ARIMA-based study

**DOI:** 10.3389/fmicb.2025.1674529

**Published:** 2025-09-25

**Authors:** İpek Koçer, Hadiye Demirbakan, Ahmet Aktaş

**Affiliations:** ^1^Department of Medical Microbiology, Faculty of Medicine Department SANKO University, Gaziantep, Türkiye; ^2^İstanbul Provincial Health Directorate, İstanbul Public Health Laboratory, İstanbul, Türkiye

**Keywords:** co-infection, multiplex real-time PCR, respiratory viruses, SARS-CoV-2, ARIMA forecasting

## Abstract

**Introduction:**

Respiratory viruses are the leading cause of acute respiratory tract infections in both children and adults, with significant morbidity and healthcare burden. Before the COVID-19 pandemic, these pathogens typically exhibited predictable seasonal circulation patterns. However, the pandemic markedly disrupted this seasonality, leading to reduced viral detection during lockdowns and unusual peaks in subsequent periods.

**Objective:**

This study aimed to identify respiratory pathogens in LRTI patients using multiplex PCR and to assess changes in virus distribution during and after the COVID-19 pandemic across age groups.

**Methods:**

A total of 748 nasopharyngeal swab samples (one per patient) collected between March 2020 and November 2024 were retrospectively analyzed using the QIAstat-Dx Respiratory Panel, a multiplex PCR assay de tecting 19 respiratory viruses including SARS-CoV-2, influenza viruses, respiratory syncytial virus (RSV), and Rhinovirus/Enterovirus. Statistical analyses, including multivariate logistic regression, assessed viral positivity predictors. Additionally, the Autoregressive Integrated Moving Average (ARIMA) time-series model was used to evaluate trends and predict respiratory virus activity through 2027.

**Results:**

The cohort comprised 60.4% males and 39.6% females, with 14.4% pediatric (0–18 years) and 85.6% adult patients. Respiratory viruses were detected in 43.6% of samples, with significantly higher positivity in children (71.5%) compared to adults (40%) (*p* < 0.01). SARS-CoV-2 dominated during the pandemic (65.5% of positive cases), whereas post-pandemic viral circulation shifted toward other pathogens, notably Rhinovirus/Enterovirus (71.5%). Co-infections occurred more frequently in children (14.1%) than adults (2.7%) (*p* < 0.001). RSV re-emerged in late 2022 but was undetected in 2024, while influenza activity increased notably in early 2024. Multivariate analysis identified pediatric age as a strong independent predictor of viral positivity (OR: 3.68; 95% CI: 2.25–6.03).

**Discussion:**

Following the relaxation of public health measures, there was a marked resurgence of non- SARS-CoV-2 respiratory viruses, particularly in children, indicating a possible shift in viral epidemiology. These findings emphasize the critical need for ongoing surveillance and targeted interventions, especially in pediatric populations, to mitigate future respiratory viral disease burdens.

## 1 Introduction

Respiratory viruses represent a significant global health burden, causing a spectrum of illnesses ranging from mild upper respiratory tract infections to severe lower respiratory tract infections (LRTIs), including pneumonia (Safiri et al., 2022). Viral pathogens constitute the predominant etiologic agents of LRTIs across both adult and pediatric populations (Monto, 2002). Epidemiological patterns of LRTIs demonstrate marked seasonal variability across different geographic regions and are influenced by the diagnostic methodologies employed to identify causative microorganisms (Johansson et al., 2010).

The COVID-19 pandemic has notably altered the epidemiological landscape of respiratory viruses (Mairi et al., 2018). This dynamic has amplified the demand for comprehensive diagnostic modalities capable of simultaneously detecting multiple respiratory viral agents. Traditional diagnostic approaches, including viral culture, pose challenges due to their labor-intensive nature and prolonged turnaround times (Krause et al., 2014). Multiplex syndromic diagnostic panels exhibit superior sensitivity and specificity compared to conventional methods, allowing detection of viral nucleic acids even at low viral loads and enabling identification of viral co-infections due to their expanded pathogen panels (Fyles et al., 2024).

This study aimed to identify respiratory pathogens in patients with LRTI using multiplex PCR and to assess changes in virus distribution during and after the COVID-19 pandemic across age groups. To anticipate future epidemiological trajectories, we used an AutoRegressive Integrated Moving Average (ARIMA) model to forecast viral trends through 2027. This integrative approach is intended to yield critical insights into the long-term epidemiological consequences of the COVID-19 pandemic and to inform strategic public health planning and preparedness efforts.

## 2 Materials and methods

### 2.1 Study design

This study retrospectively analyzed 748 nasopharyngeal swabs from patients with suspected LRTI, collected at Sanko University Hospital between March 2020 and November 2024. Multiplex PCR testing was used in selected clinical settings such as emergency units, ICUs, pediatric services, hematology-oncology, and transplant wards. PCR results were evaluated to identify viral pathogens in positive cases. Patients were stratified into pediatric (0–18 years) and adult (>18 years) cohorts, and the study timeframe was divided into pandemic (March 2020–December 2021) and post-pandemic (January 2022–November 2024) periods based on the World Health Organization’s delineation of the COVID-19 pandemic timeline. Nasopharyngeal and anterior nasal swab samples were collected from patients of all ages presenting with acute respiratory infection symptoms within the previous seven days. Physicians ordered multiplex PCR testing based on clinical features such as fever, cough, sore throat, nasal congestion or discharge, shortness of breath, chills, myalgia, headache, and fatigue. Symptoms were further classified as respiratory or systemic for analysis. Patients who had recently received antiviral therapy or were enrolled in other clinical protocols were excluded.

### 2.2 Laboratory analysis

Detection of respiratory viruses was performed using the QIAstat-Dx® Respiratory Panel Test (Qiagen, Germany), a multiplex real-time PCR assay. The panel simultaneously detects 19 viral and 3 bacterial targets, including Adenovirus; Coronaviruses 229E, HKU1, NL63, and OC43; Human metapneumovirus A/B; Influenza A (including subtypes H1N1/2009, H3, and H1), Influenza B, Parainfluenza viruses 1–4; Rhinovirus/Enterovirus; Respiratory Syncytial Virus A/B (RSV-A/B); SARS-CoV-2; *Bordetella pertussis; Legionella pneumophila*; and *Mycoplasma pneumoniae* (Visseaux et al., 2020).

### 2.3 Statistical analysis

Descriptive statistics were used to summarize demographic data and overall viral positivity rates. Chi-square tests assessed associations between categorical variables, and trend analyses examined temporal changes. Multivariate logistic regression analysis was used to identify independent predictors of viral positivity, with age, gender, and time period as covariates. Multinomial and binary logistic regression models were constructed to explore associations with specific pathogens and co-infections. Adjusted odds ratios (ORs) and corresponding 95% confidence intervals (CIs) were reported. To analyze and forecast pathogen-specific trends, ARIMA time-series models were applied to monthly case counts from January 2020 to December 2024. Separate models were built for SARS-CoV-2, Rhinovirus/Enterovirus, and RSV, with optimal parameters selected using the Akaike Information Criterion. Forecasts for January 2025–December 2027 included point estimates and 95% CIs. All statistical analyses were performed using IBM SPSS Statistics v.27 and R software v. 4.2. Data visualizations, including line plots, stacked bar charts, forest plots, and time-series forecasts with CIs, were generated using the ggplot2 package in R and the matplotlib library in Python.

### 2.4 Time series analysis and forecasting

Monthly counts of positive cases from January 2020 through December 2024 were analyzed using pathogen-specific ARIMA models for SARS-CoV-2, Rhinovirus/Enterovirus, and RSV. Prior to model fitting, data stationarity and autocorrelation structures were evaluated using appropriate statistical tests and correlograms. The optimal ARIMA model parameters were selected based on minimization of the Akaike Information Criterion (AIC). Forecasts were subsequently generated for a 36-month horizon, spanning January 2025 to December 2027. Prediction outputs include point estimates alongside 95% CIs to quantify forecast uncertainty.

## 3 Results

A total of 748 nasopharyngeal swab specimens from patients with suspected LRTI were analyzed. Among these patients, 452 (60.4%) were male and 296 (39.6%) were female. The distribution of respiratory viruses was evaluated across two age groups: pediatric patients (0–18 years) and adults (>18 years), recognizing the distinct clinical course and epidemiological profiles of viral infections between children and adults. Of the total samples, 109 (14.4%) originated from pediatric patients, while 639 (85.6%) were collected from adult patients, with a median age of 37 years (interquartile range: 26–50 years).

Using multiplex PCR, simultaneous detection of 19 respiratory viruses was performed. No pathogen was identified in 422 samples (56.4%), whereas at least one viral agent was detected in 326 samples (43.6%). The detection rate of viral pathogens was significantly higher in the pediatric group, with 71.5% (78/109) testing positive, compared to 40.0% (256/639) in the adult cohort (*p* < 0.01). While females exhibited a marginally higher positivity rate (155/296; 52.4%) compared to males (224/452; 49.6%), this difference did not reach statistical significance (*p* = 0.905).

Positive cases were stratified into two temporal cohorts—pandemic (March 2020 to December 2021) and post-pandemic (January 2022 to November 2024)—in accordance with the World Health Organization’s delineation of the COVID-19 pandemic timeline. This temporal division facilitated assessment of epidemiological shifts before and after widespread public health interventions.

**Figure 1:**
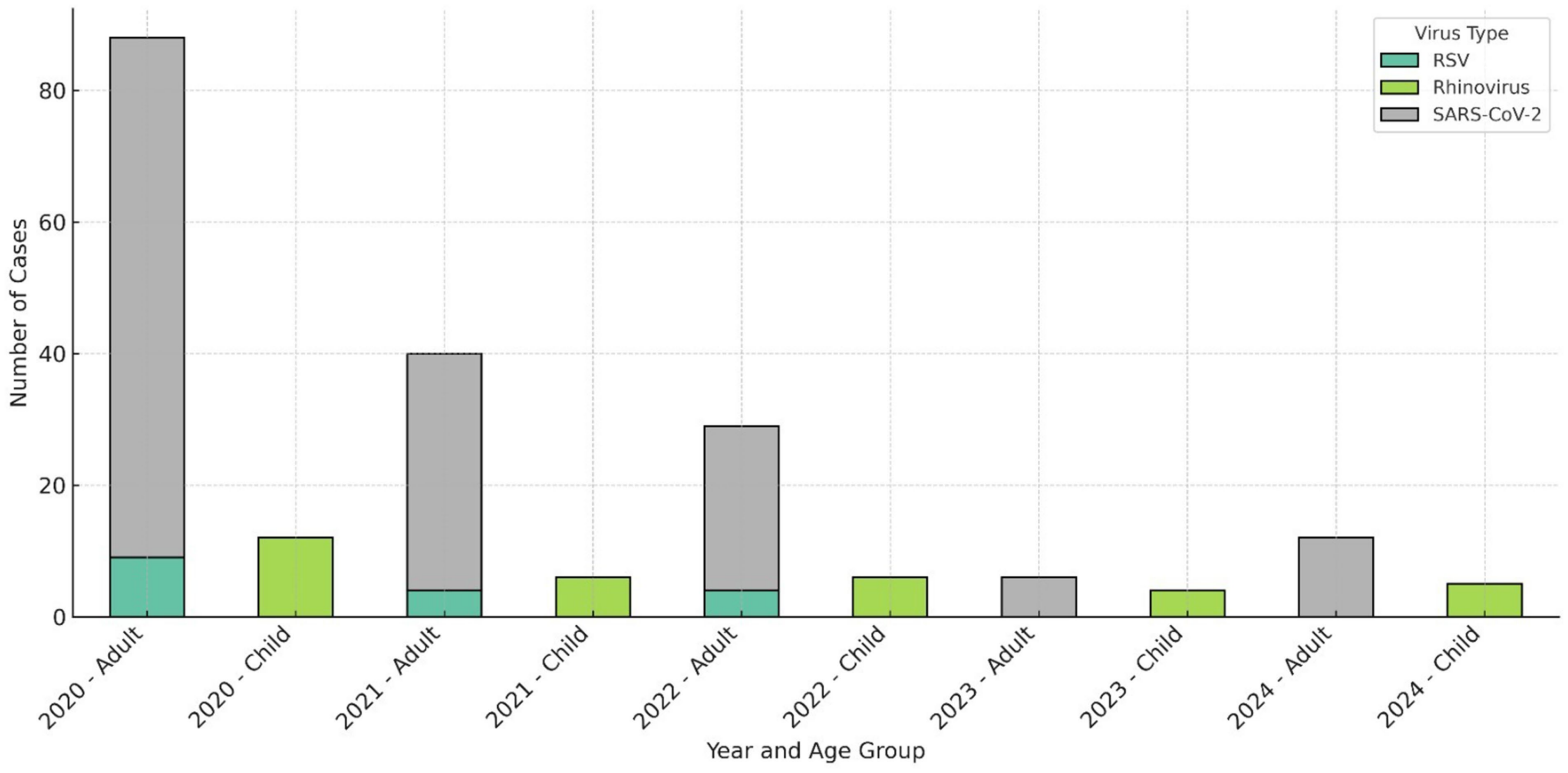
Annual distribution of predominant respiratory viruses stratified by age group. SARS- CoV-2 remained the most prevalent pathogen among adults throughout the pandemic period, whereas Rhinovirus/Enterovirus emerged as the dominant virus in the pediatric population during the post- pandemic phase. RSV activity was pronounced in 2022 but was not detected in 2024.

The annual number of respiratory virus tests and corresponding positivity rates demonstrated considerable variability. In 2020, 411 samples were analyzed with 144 (35.0%) testing positive. Testing volume decreased markedly in 2021–84 samples, among which 46 (54.8%) were positive. In 2022, the positivity rate increased further to 64.0% (48/75 positive samples), followed by a decline to 50.0% (36/72) in 2023. In 2024, testing volume increased slightly to 105, with a positivity rate of 57.1% (60 positive cases). Despite fluctuations in testing numbers during the post-pandemic period, an overall increasing trend in positivity rates was observed (*p* < 0.01). The significant association between younger age and increased viral positivity was subsequently confirmed by multivariate logistic regression analysis (Figure 1).

Among the samples in which at least one viral agent was identified during the pandemic period, SARS-CoV-2 was the predominant pathogen, detected in 65.5% (122/190) of cases. Conversely, during the post-pandemic period, 71.5% (103/144) of positive samples were attributable to non–SARS-CoV-2 viral infections, with a statistically significant difference between the two periods (*p* < 0.0001) Following SARS-CoV-2, the most frequently detected viruses during the pandemic were Rhinovirus/Enterovirus (37/190; 19.5%) and Respiratory Syncytial Virus A/B (RSV-A/B) (13/190; 6.8%).

In the post-pandemic interval (2022–2023), among samples positive for at least one viral agent, Rhinovirus/Enterovirus predominated in the pediatric population (0–18 years) at 26.5% (9/34), whereas SARS-CoV-2 remained the leading viral agent in adults (>18 years) at 46.2% (25/54).

Co-infections involving two or more pathogens were identified in 5.5% (18/326) of all positive samples. The co-infection rate was significantly higher in children (0–18 years), at 14.1% (11/78), compared to 2.7% (7/256) in adults (*p* < 0.001). Among pediatric co-infections, Rhinovirus/Enterovirus was the most frequent co-infecting agent, accounting for 61.1% (11/18) of co-infection cases. In adults, SARS-CoV-2 was the most commonly involved virus in co-infections. The detailed distribution of co- infecting pathogens stratified by age and gender is summarized in Table 1.

**Table 1:** Distribution of co-infection cases by viral pathogens, age group, and gender.

Viral pathogens	Pediatric (0–18 years)		Adult (>18 years)		Total *n* (%)
	Male (%)	Female (%)	Male (%)	Female (%)	
SARS-CoV-2 + Rhino/Enterovirus	1	1	2	–	4 (22.2)
SARS-CoV-2 + *M. pneumoniae*	–	–	1	–	1 (5.5)
SARS-CoV-2 + Parainfluenza virus	–	1	1	1	3 (16.7)
Rhino/Enterovirus + Parainfluenza virus	1	–	–	–	1 (5.5)
Rhino/Enterovirus + Adenovirus	1	–	–	–	1 (5.5)
Rhino/Enterovirus + Bocavirus	1	1	–	–	2 (11.1)
Rhino/Enterovirus + hMPV A + B	–	–	1	–	1 (5.5)
Rhino/Enterovirus + RSV-A/B	1	–	–	–	1 (5.5)
Rhino/Enterovirus + *B. pertussis*	-	1	–	–	1 (5.5)
Influenza A + RSV-A/B	–	–	–	1	1 (5.5)
Adenovirus + Bocavirus	1	–	–	–	1 (5.5)
Adenovirus + RSV-A/B		1			1 (5.5)
Total *n* (%)	6 (33.3)	5 (27.7)	5 (27.7)	2 (11.1)	18 (100)

**Figure 2:**
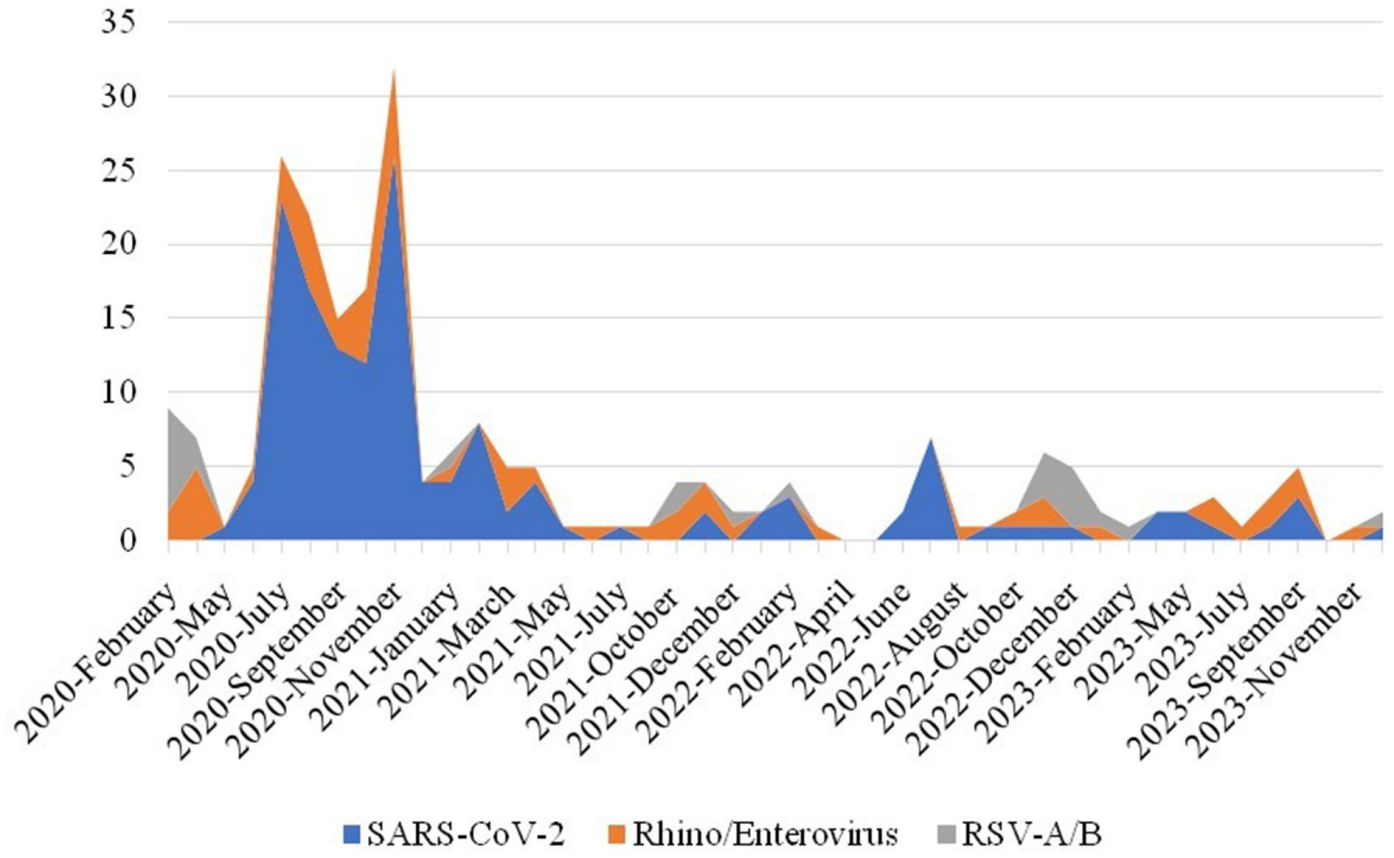
Monthly distribution of the most frequently detected respiratory viral agents during 2020–2023.

When examining the temporal distribution of positive results, November 2020 exhibited the highest positivity rate at 60.3% (32/53). SARS-CoV-2 activity was sustained continuously throughout 2020, peaking in November with a positivity rate of 49% (26/53). In terms of seasonal variation among the most prevalent viral agents, Rhinovirus/Enterovirus circulated steadily throughout 2020, with peak incidences of five cases in October and six cases in November. RSV-A/B cases declined markedly during the pandemic period but demonstrated resurgence in late 2022, reaching a peak of three cases in November and four cases in December. Notably, RSV was undetected in 2024. The seasonal distribution patterns of SARS-CoV-2, Rhinovirus/Enterovirus, and RSV-A/B are illustrated in Figure 2.

Influenza virus activity remained limited during the pandemic, with a single positive case detected in 2020 (7.1%). However, influenza positivity increased in the post-pandemic interval (2022–2023), with eight positive samples accounting for 9.5% of cases. In the first quarter of 2024, influenza virus activity peaked substantially, comprising 30% (18/60) of positive detections.

The ARIMA model demonstrated an adequate fit, with no significant residual autocorrelation as indicated by the Ljung-Box test (*p* > 0.05) (Supplementary Table 2). Forecasted monthly case counts for the period 2025–2027 ranged between 15 and 26, with anticipated peaks occurring in late winter and early spring. These projections suggest periodic surges in viral detections consistent with established seasonal patterns of respiratory virus circulation.

The forecasting outcomes reveal the potential for recurring seasonal peaks in respiratory virus activity despite overall post-pandemic normalization. Based on the three primary respiratory pathogens analyzed, distinct temporal dynamics and varying degrees of forecast uncertainty were observed. SARS- CoV-2 exhibited the greatest variability in predicted case numbers, reflecting the potential for significant fluctuations and ongoing risk of future increases. In contrast, Rhinovirus/Enterovirus demonstrated relatively stable forecasted case counts with narrow CIs, indicative of predictable and seasonally consistent circulation. RSV A/B showed a modest and steady trend with less pronounced variability compared to SARS-CoV-2.

**Figure 3:**
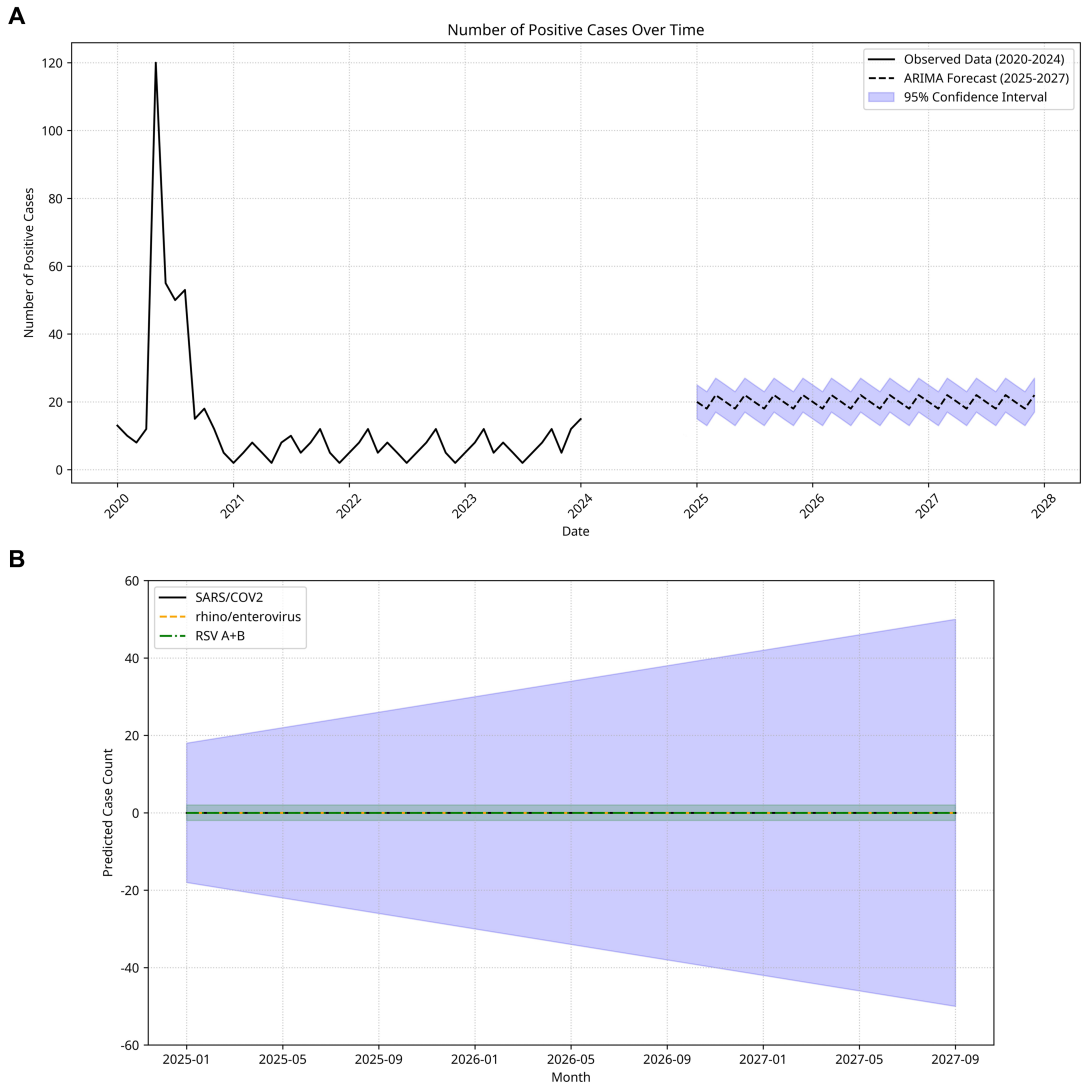
**(A)** Temporal distribution of respiratory virus-positive cases and ARIMA model forecasts (2020–2027). The solid black line represents observed cases, showing a sharp peak in early 2020 corresponding to the onset of the pandemic, followed by a rapid decline and stabilization at relatively low levels in subsequent years. The dashed black line indicates predicted values for 2025–2027, and the shaded blue area represents the 95% confidence interval of the forecast. Model predictions suggest that the number of positive cases will remain relatively stable in the coming years, with mild seasonal fluctuations, and are unlikely to return to the extreme levels observed during the initial pandemic wave. **(B)** Monthly Estimated Case Counts and 95% Confidence Intervals for Three Major Respiratory Pathogens (SARS-CoV-2, Rhinovirus/Enterovirus, RSV A + B) (January 2025–December 2027). These pathogens were selected for detailed projection owing to their clinical relevance. The solid black, dashed orange, and dashed–dotted green lines represent the predicted case counts for each virus, while the shaded blue area indicates the 95% confidence interval of the forecasts. Predictions for all three pathogens remain close to zero throughout the forecast period, suggesting very low or negligible circulation under the current model assumptions. However, the wide confidence intervals highlight considerable uncertainty, particularly given the potential for seasonal variation, emerging variants, or unanticipated epidemiological changes. These findings emphasize the need for cautious interpretation of long-term forecasts and underline the importance of continuous surveillance to capture deviations from model expectations.

To enhance visual clarity and minimize overlap of uncertainty bands, 95% CIs were employed in the figures, providing a balance between interpretability and representation of model uncertainty. These long-term projections offer critical insights for public health planning by informing anticipation of seasonal respiratory virus activity, optimizing allocation of healthcare resources, and guiding targeted interventions in the post-pandemic context. The findings emphasize the necessity for ongoing surveillance and preparedness to mitigate potential surges, particularly during winter months. ARIMA- based forecasting thus serves as a valuable tool in strategic public health decision-making (Figure 3).

**Figure 4:**
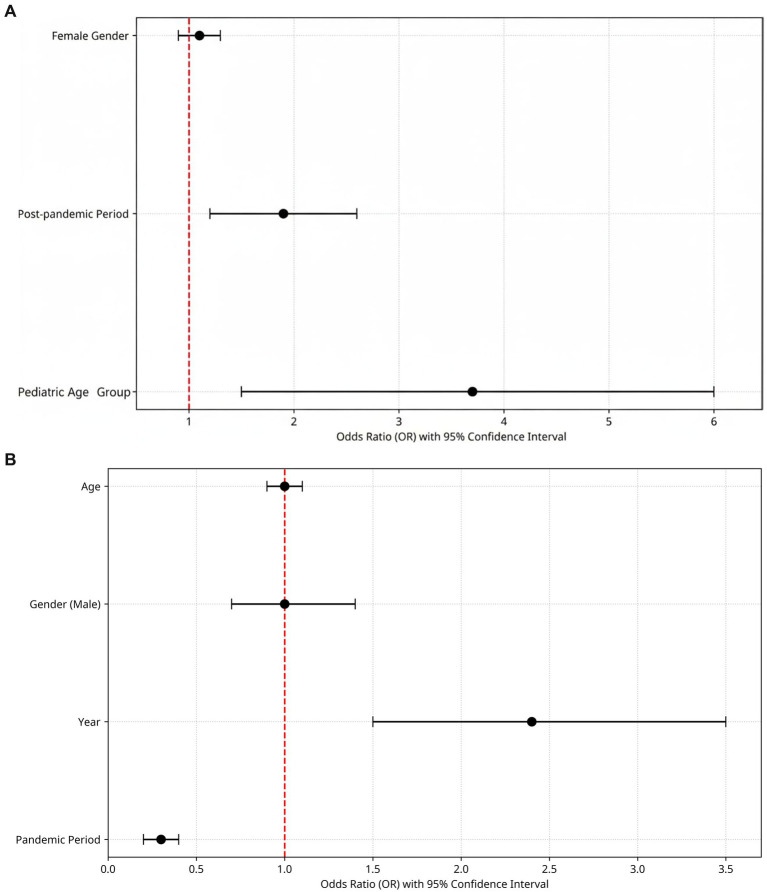
**(A)** Predictors of viral positivity: a forest plot of multivariate logistic regression analysis. Forest plot of multivariate logistic regression analysis for predictors of respiratory viral test positivity. Black circles indicate odds ratios (OR), horizontal lines represent 95% confidence intervals (CI), and the vertical red dashed line denotes the null value (OR = 1). Female gender was not significantly associated with viral positivity (OR = 0.99, 95% CI: 0.71–1.39). In contrast, the post-pandemic period was independently associated with increased odds of positivity (OR = 1.92, 95% CI: 1.20–3.07), and the pediatric age group (0–18 years) showed the strongest effect, with markedly higher odds of positivity (OR = 3.68, 95% CI: 2.25–6.03, *p* < 0.001). **(B)** The logistic regression model of PCR positivity. The red dashed vertical line indicates the null value (OR = 1.0). Dots represent estimated ORs, while horizontal lines indicate 95% CIs. Children (0–18 years) had significantly higher odds of testing positive (OR: 3.68, 95% CI: 2.25–6.03, *p* < 0.001). Being in the post-pandemic period also increased positivity risk (OR: 1.92, *p* < 0.01), whereas gender had no significant effect.

Logistic regression analysis showed a negative association between viral positivity and the pandemic period. However, due to complete dissociation, the odds ratio and confidence interval could not be estimated. This reflects a significant decrease in respiratory virus detection during this period. Conversely, calendar year was positively associated with viral positivity (OR = 1.404, 95% CI: 1.201–1.640), suggesting a gradual increase in positivity rates over time. Gender did not exhibit a statistically significant effect (OR = 0.993, 95% CI: 0.709–1.389), as the confidence interval included unity. Age demonstrated a modest but statistically significant inverse relationship with viral positivity (OR = 0.977, 95% CI: 0.969–0.985), indicating that younger individuals had a slightly higher likelihood of testing positive (Figure 4).

## 4 Discussion

This study retrospectively analyzed respiratory virus panel multiplex real-time PCR test results obtained during the COVID-19 pandemic and the subsequent post-pandemic period. The findings are expected to inform and enhance strategies aimed at controlling respiratory viral pathogens.

Molecular diagnostic techniques characterized by high sensitivity and rapid turnaround times play a pivotal role in the early detection of respiratory viral pathogens (Hanson et al., 2020). Syndromic panels offer substantial advantages in identifying viral etiology, including simultaneous detection of multiple pathogens, user-friendly operation, high sensitivity and specificity, and rapid result availability (Leber et al., 2020; Huang et al., 2018). The global magnitude and rapid dissemination of COVID-19 underscored the critical need for rapid and comprehensive diagnostic modalities in the management of viral diseases (Maataoui et al., 2021).

This study evaluated patients presenting with LRTI symptoms who tested positive for respiratory viruses by multiplex PCR on nasopharyngeal swabs between 2020 and 2024. The data were stratified into two temporal periods: the initial phase of the COVID-19 pandemic and gradual normalization (March 2020 to December 2021) and the post-pandemic period (January 2022 to November 2024). The syndromic panel was predominantly utilized for high-risk populations such as intensive care unit patients, trans-plant recipients, and pediatric emergency cases. The detection rate of at least one viral agent during the pandemic period and its first year (2020–2021) was 38.3%, which increased to 55.7% in the post-pandemic interval (2022–2023), further rising to 57.1% in 2024. These findings align with previous studies reporting a marked decline in respiratory virus activity during the pandemic and lockdown phases, followed by a resurgence beginning in 2021 (Chow et al., 2023). The relaxation of public health measures and return to routine social life were accompanied by a marked rise in respiratory infections. Importantly, positivity rates continued to increase despite yearly variations in testing volume, indicating a true post-pandemic resurgence rather than a testing artifact and highlighting the need for ongoing surveillance. A recent study investigating respiratory infections in children reported a significant increase in complicated respiratory tract infections during the 2022/2023 winter season compared to both pre-pandemic and pandemic years, following a notable decline in the first pandemic winter (Metz et al., 2024). In our cohort, SARS-CoV-2 was the predominant pathogen detected during the COVID-19 pandemic and the initial normalization period, accounting for 64.2% (n = 190) of samples positive for at least one viral agent. Coinfections involving Rhinovirus/Enterovirus were identified in four cases, and *Mycoplasma pneumoniae* was detected in one case. Although the prevalence of non– COVID-19 respiratory viruses declined significantly during the pandemic, their detection rate increased markedly from 34.5% during the pandemic to 66.6% in the post-pandemic period.

Logistic regression analysis confirmed a strong negative association between the pandemic period and viral positivity, and a positive association with calendar year, indicating a gradual resurgence of respiratory viruses after peak restrictions. Younger age was independently linked to higher positivity, reflecting pediatric vulnerability and likely reduced prior exposure during lockdowns. Although females showed slightly higher positivity than males (52.4% vs. 49.6%), the difference was not significant (*p* = 0.905), and gender was not a significant predictor in the model. These findings suggest that respiratory virus detection rates were not substantially influenced by gender in this cohort.

Post-pandemic, Rhinovirus/Enterovirus was the most common virus in children (20%; n = 6), while SARS-CoV-2 predominated in adults (46.2%; n = 25), likely due to the environmental resilience and prolonged shedding of non-enveloped viruses. During the pan-demic, mitigation measures suppressed many respiratory viruses, but their easing led to broader viral circulation (Madaniyazi et al., 2022).

The observed monthly trends also highlight distinct seasonal patterns for specific viruses. SARS- CoV-2 activity peaked in November 2020, while RSV re-emerged in late 2022 but was notably absent in 2024. Rhinovirus/Enterovirus showed consistent year-round activity with typical seasonal peaks. These patterns support the hypothesis of altered but gradually normalizing seasonality of respiratory viruses post-pandemic. A study conducted in Türkiye reported a significant decline in the frequency of viral infections other than SARS-CoV-2 during the pandemic, followed by an increase in positivity rates for other respiratory viruses from 9.4 to 39.5% during the normalization period (Alt et al., 2022), find ings that are corroborated by our study. This resurgence of respiratory pathogen infections in the post- pandemic phase is hypothesized to result not only from the relaxation of public health precautions but also from diminished population immunity caused by reduced exposure to respiratory pathogens during the COVID-19 pandemic, thereby increasing in-dividual susceptibility (Gan et al., 2024). Zhang et al. (2023) reported a decrease in the positivity rate of other respiratory viral pathogens from 61.6% in the pre-pandemic period to 40.3% during the COVID-19 pandemic. Consistent with these findings, Influenza A positivity was detected in only a single case during the 2020 pandemic period in our study, but activity increased starting in late 2021 and persisted throughout the 2022–2023 winter seasons. According to the Weekly Influenza Surveillance Report from the Ministry of Health of the Republic of Türkiye Ministry of Health, General Directorate of Public Health (2023), there was a 99% reduction in influenza infections during the 2020–2021 season, with a peak of 32.5% positivity among respiratory samples from symptomatic patients observed in the last weeks of 2022. This pattern aligns with reports from Australia and the United States, which documented delayed influenza circulation attributed to prolonged COVID-19 mitigation strategies (Razanajatovo et al., 2022).

Multiplex PCR testing facilitates simultaneous detection of multiple pathogens in a single respiratory sample, thereby reducing unnecessary antibiotic use and enabling targeted antimicrobial therapy by identifying co-infections, which are more prevalent among children (Elnifro et al., 2000). In our cohort, the overall co-infection rate was 6.5%, with significantly high-er rates in pediatric patients (15%) compared to adults (3.5%) (*p* < 0.001). Co-infections are more frequent during conditions conducive to respiratory viral transmission such as winter months, indoor settings, schools, daycare centers, and nursing homes, with the highest rates observed in children under five years of age (Mandelia et al., 2021). Zhang et al. (2023) and Le Glass et al. (2021) similarly identified Rhinovirus/Enterovirus as the most common viral agent in co-infections, consistent with our results. Studies conducted in Türkiye during the pandemic also identified Rhinovirus/Enterovirus as the predominant co-infecting virus (Alp et al., 2022).

Although multiplex PCR-based methods provide a valuable tool for the rapid and sensitive detection of bacterial pathogens, they may, in some cases, reflect colonization rather than true infection, which represents a fundamental limitation of multiplex PCR. Moreover, not all potential bacterial pathogens are included in every multiplex PCR panel; therefore, conventional bacterial cultures remain important for diagnostic purposes, particularly in patients with acute respiratory infection symptoms after the pandemic. While multiplex PCR can detect key resistance genes, its ability to provide detailed antimicrobial susceptibility profiles is limited. Combining multiplex PCR with conventional microbiological culture techniques can enhance diagnostic yield and allow more accurate assessment of co-infections. During the pandemic, bacterial culture testing in COVID-19 patients was rarely requested and performed at low rates. Low rates of microbiological testing can lead to overestimation or underestimation of bacterial co-infections, which may limit the accurate evaluation of co-infections associated with COVID-19. Our respiratory panel included three bacterial pathogens; however, bacterial cultures were not obtained from all patients. In a separate study conducted during the pandemic, *Streptococcus pneumoniae* co-infection was identified in 11 patients with confirmed COVID-19 pneumonia, underscoring the importance of monitoring bacterial co-infections in viral lung infections (Le Glass et al., 2021). The clinical significance of bacterial culture in patients with SARS-CoV-2 infection is emphasized due to the potential for bacterial co-infection to worsen prognosis.

In this study, we applied the ARIMA model to forecast the temporal dynamics of key respiratory viruses from 2025 to 2027, utilizing multiplex PCR data collected between 2020 and 2024. ARIMA is a well-established time series modeling approach extensively employed in infectious disease epidemiology due to its transparency, adaptability, and relatively modest data requirements (Zheng et al., 2024). Our pathogen-specific forecasts revealed distinct temporal patterns: SARS-CoV-2 exhibited broad prediction intervals indicative of ongoing epidemiological volatility, whereas Rhinovirus/Enterovirus demonstrated consistent seasonal trends with narrow confidence intervals. RSV A + B showed moderate variability, suggesting a partial re-establishment of pre-pandemic seasonality. Nonetheless, ARIMA models possess inherent limitations, particularly their inability to explicitly account for sudden external perturbations such as the emergence of novel viral variants or abrupt shifts in public health interventions (Wang et al., 2022; Zhang et al., 2020). Despite these constraints, the incorporation of ARIMA-based forecasts into routine respiratory virus surveillance systems may substantially enhance early warning capabilities and inform public health preparedness, especially for pathogens exhibiting re-emergent or unpredictable seasonal dynamics.

While multiplex PCR testing is generally available in clinical settings such as emergency units, ICUs, pediatric services, hematology-oncology, and transplant wards, the population in our study primarily consisted of outpatients. The data presented here therefore reflect the use of nucleic acid amplification-based technologies (NAATs) for respiratory pathogen detection in an ambulatory care population, where some policy uncertainties regarding testing may exist. This study has several limitations. First, it was conducted in a single center and included only symptomatic patients, which may limit the generalizability of the findings and potentially lead to an underestimation of the true prevalence of infections. Therefore, future multicenter studies including both symptomatic and asymptomatic populations are warranted to provide more comprehensive data. Second, the absence of bacterial culture data may have led to underestimation of bacterial co-infections, which are clinically important for guiding antimicrobial therapy and patient management decisions. Third, the use of nasopharyngeal specimens alone may have limited detection of lower respiratory tract infections, and post-infectious viral shedding or asymptomatic colonization may have led to overestimation of infection and co-infection rates. Fourth, seasonal variations and changes in healthcare-seeking behavior during the pandemic may have affected viral prevalence estimates. Additionally, although ARIMA modeling provided useful time-series forecasts, it is limited by its inability to account for unforeseen external factors, such as the emergence of novel viral variants or changes in public health interventions. This methodological limitation is particularly relevant for SARS-CoV-2, whose evolutionary dynamics are unpredictable compared to other respiratory viruses, resulting in wider prediction intervals and higher epidemiological uncertainty. Consequently, prediction intervals were wider for SARS-CoV-2, reflecting the higher uncertainty and epidemiological volatility of this pathogen. Future studies may improve long-term projections by incorporating external variables such as variant prevalence, vaccination coverage, mobility indices, or hybrid modeling approaches. Despite these limitations, a major strength of our study is the clear demonstration of the advantages of multiplex PCR approaches over single-pathogen tests for detecting bacterial, viral, and fungal respiratory pathogens. This is particularly relevant in the context of a global pandemic, where diagnostic resources may be limited. Our findings highlight the potential role of expanded respiratory pathogen panels in guiding treatment decisions and improving outcomes, especially in patients with lower respiratory tract infections who are at high risk of complications. Future studies should adopt prospective, multicenter designs including high-risk populations and detailed follow-up to further evaluate the clinical utility of extended multiplex PCR panels and their impact on patient management.

## 5 Conclusion

Multiplex PCR enables rapid and accurate detection of respiratory viruses, including co- infections. This study analyzes virus circulation during and after the COVID-19 pandemic, showing a notable resurgence of Rhinovirus/Enterovirus and influenza following the lifting of restrictions. Pediatric patients were more affected, with higher co-infection rates. ARIMA modeling revealed seasonal patterns and predicted future peaks, highlighting the need for ongoing surveillance. These trends may indicate a lasting shift in viral epidemiology, warranting further studies that include asymptomatic cases and environmental factors. Long-term multiplex PCR surveillance combined with time-series modeling is valuable for monitoring viral epidemiology and guiding timely public health interventions.

## Data Availability

The original contributions presented in the study are included in the article/Supplementary material, further inquiries can be directed to the corresponding author.
