# Supplementary material for: Temporal dynamics and forecasting of respiratory viral infections during and after the SARS-CoV-2 pandemic (2020–2027): a multiplex PCR and ARIMA-based study
Source: Front Microbiol. 2025 Sep 25;16:1674529. doi: 10.3389/fmicb.2025.1674529 (PMC12507870; doi:10.3389/fmicb.2025.1674529)
Supplement: Supplementary file 1 [file Data_Sheet_1.docx]

|  | Pandemic period  (2020-2021) | | | | Post-pandemic period  (2022-2023) | | | | Post-pandemic period  (2024) | | | | | |
| --- | --- | --- | --- | --- | --- | --- | --- | --- | --- | --- | --- | --- | --- | --- |
| Viral pathogens | **0-18 age**  **n (%)** | | **>18 age**  **n (%)** | | **0-18 age**  **n (%)** | | **>18 age**  **n (%)** | | **0-18 age**  **n (%)** | | | **>18 age**  **n (%)** | |  |
|  | Male | Female | Male | Female | Male | Female | Male | Female | Male | Female | | Male | Female |  |
| SARS-CoV-2 | 4 (2.1) | 3 (1.6) | 79 (41.6) | 36 (18.9) | - | 3 (3.6) | 10(5.3) | 15 (7.9) | 1(1.7) | - | | 6 (10) | 6 (10) |  |
| Rhino/Enterovirus | 12 (6.3) | 3 (1.6) | 14 (7.4) | 8 (4.2) | 2(2.4) | 4 (4.8) | 6 (7.2) | 2 (2.4) | 1(1.7) | | - | 4 (6.7) | - |  |
| RSV-A/B | - | - | 9 (4.7) | 4 (2.1) | 2(7.2) | 5(5.9) | - | 4 (4.8) | - | | - | - | - |  |
| HCoV | - | 1 (0.5) | 3 (1.6) | 3 (1.6) | 3(3.6) | 1 (1.2) | 4 (4.8) | 3 (3.6) | - | | - | 2 (3.3) | 3 (5) |  |
| Influenza A | 1 (0.5) | - | 2 (1) | 1 (0.5) | 2(2.4) | 2(2.4) | 3(3.6) | 2(2.4) | - | | - | 8 (13.3) | 9 (15) |  |
| Bocavirus | 3 (1.6) | 3(1.6) | 1(0.5) | - | 1(1.2) | 1(1.2) | - | - | - | | - | 1 (1.7) | - |  |
| Parainfluenza | 1(0.5) | 1(0.5) | 1(0.5) | - | 2(2.4) | 2 (2.4) | 4 (4.8) | 4 (4.8) | - | | - | 2 (3.3) | - |  |
| Adenovirus | 1(0.5) | - | - | - | 2(2.4) | 1 (1.2) | - | - | 1 (1.7) | | - | - | - |  |
| HMPV A+B | - | 1 (0.5) | - | - | - | 2(2.4) | 1(1.2) | - | - | | - | 1 (1.7) | 1 (1.7) |  |

**Supplement Table 1.** Distribution of viral respiratory pathogens during and after the COVID-19 pandemic.
