# Supplementary material for: Temporal dynamics and forecasting of respiratory viral infections during and after the SARS-CoV-2 pandemic (2020–2027): a multiplex PCR and ARIMA-based study
Source: Front Microbiol. 2025 Sep 25;16:1674529. doi: 10.3389/fmicb.2025.1674529 (PMC12507870; doi:10.3389/fmicb.2025.1674529)
Supplement: Supplementary file 2 [file Data_Sheet_2.docx]

## Supplement Table 2. ARIMA Forecast Summary for Major Respiratory Viruses (2025–2027)

| Year | SARS-CoV-2 (95% CI) | Rhino/Enterovirus (95% CI) | RSV (95% CI) |
| --- | --- | --- | --- |
| 2025 | 0.8 (–12.0 to 14.2) | 1.3 (–2.9 to 6.1) | 1.5 (–5.1 to 8.0) |
| 2026 | 0.9 (–10.8 to 13.5) | 1.4 (–3.1 to 6.4) | 1.6 (–4.8 to 8.4) |
| 2027 | 0.9 (–11.2 to 13.9) | 1.5 (–3.0 to 6.8) | 1.7 (–4.6 to 8.9) |
